# 

**Published:** 1892-08

**Authors:** 


### CONTENTS.

ORIGINAL COMMUNICATIONS—

What Shall We Do with the Uterus after Abortions? By K. P. Moore, M. D., Macon, Ga. .... 321
Adenoid Vegetations of the Pharynx a Frequent Cause of Deafness in Children. Their Removal. By S. Latimer Phillips, M. D., Savannah, Ga. .... 329
The Cure of Cancer by Mattei-ism. By M. Louis Boucher. Normandie Medicale, 1st April, 1892. Translated from the French, by Thomas H. Manley, M. D., New York .... 334
The Relation between Skin Diseases and the General Health. By M. B. Hutchins, M. D., Atlanta, Ga. .... 338
A Case of Occlusion of the Os Uteri. By William L. Davis, M. D., Albany, Ga. .... 342
A Case of Intestinal Injury—Recovery. By F. C. Johnston, M. D., Richland, Ga. .... 344

SOCIETY REPORTS—

Clinical Society of Maryland .... 346

CORRESPONDENCE—

A Peculiar Case of Idiosyncrasy .... 348
New York Letter .... 349

EDITORIAL—

The Present Status of the Medical Colleges of Georgia .... 353
Higher Medical Education .... 356

SELECTIONS .... 358
MEDICAL ITEMS .... 379
BOOK REVIEWS .... 382
INDEX TO ADVERTISEMENTS .... xii
ITEMS OF INTEREST .... xi

